# Molecular Verification of Bloom-forming *Aphanizomenon flos-aquae* and Their Secondary Metabolites in the Nakdong River

**DOI:** 10.3390/ijerph15081739

**Published:** 2018-08-13

**Authors:** Hae-Kyung Park, Mi-Ae Kwon, Hae-Jin Lee, Jonghee Oh, Su-Heon Lee, In-Soo Kim

**Affiliations:** 1 Nakdong River Environment Research Center, National Institute of Environmental Research, Goryeong 40103, Korea; kma81@daum.net (M.-A.K.); hjlee76@korea.kr (H.-J.L.); factork@korea.kr (I.-S.K.); 2 School of Applied Biosciences, Kyungpook National University, Daegu 41566, Korea; jheeoh09@gmail.com (J.O.); suheon@knu.ac.kr (S.-H.L.)

**Keywords:** *Aphanizomenon flos-aquae*, internal transcribed spacer (ITS), 16S rRNA, cyanobacterial toxin biosynthesis genes, Nakdong River

## Abstract

*Aphanizomenon* spp. have formed harmful cyanobacterial blooms in the Nakdong River during spring, autumn, and now in winter, and the expansion of blooming period and area, associated with the global warming is predicted. The genus *Aphanizomenon* has been described to produce harmful secondary metabolites such as off-flavors and cyanotoxins. Therefore, the production of harmful secondary metabolites from the *Aphanizomenon* blooms in the Nakdong River needs to be monitored to minimize the risk to both water quality and public health. Here, we sampled the cyanobacterial blooms in the Nakdong River and isolated ten *Aphanizomenon* strains, morphologically classified as *Aphanizomenon flos-aquae* Ralfs ex Bornet et Flahault 1888. Phylogenetic analysis using 16S rRNA and internal transcribed spacer (ITS) region nucleotide sequences confirmed this classification. We further verified the harmful secondary metabolites-producing potential of *A. flos-aquae* isolates and water samples containing cyanobacterial blooms using PCR with specific primer sets for genes involved in biosynthesis of off-flavor metabolites (geosmin) and toxins (microcystins, saxitoxins and cylindrospermopsins). It was confirmed that these metabolite biosynthesis genes were not identified in all isolates and water samples containing only *Aphanizomenon* spp. Thus, it is likely that there is a low potential for the production of off-flavor metabolites and cyanotoxins in *Aphanizomenon* blooms in the Nakdong River.

## 1. Introduction

Harmful cyanobacterial blooms (cyanoHAB) are one of the largest global issues regarding the water quality of fresh waters. They cause various water utilization problems, including reduction of the value of drinking water sources by causing odors or producing toxic substances harmful to livestock and people and by interfering with recreational activities through formation of cyanobacterial scums on the water surface. It has been predicted that the amount and duration of cyanoHAB will increase along with recent climate changes [1,2]. Moreover, global warming is associated with the apparent spread of some nostocalean cyanobacteria from tropical to temperate latitudes [3,4]. 

The Nakdong River is the second largest river in Korea, originating from Hwangji of the Taebaek Mountains and running 525 km to the South Sea, covering 23,817 km^2^ of basin area. In addition to serving as the agricultural and industrial water source, this river serves as the drinking water source for the 13 million people who live nearby [5]. However, large cities as well as various agricultural industrial complexes in the basin have polluted the Nakdong River, and there have been reports of cyanoHAB in the downstream section of the Nakdong River since the 1990s [6,7]. After the construction of the eight weirs along the river in 2012, there has been cyanoHAB formation every summer [8,9]. In addition, cyanoHAB, which has been known to only occur during seasons with high water temperature, was recently observed to persist even during the winter, and the nostocalean cyanobacteria, *Aphanizomenon* spp., were the most dominant [10]. *Aphanizomenon* spp. are characterized by high ecological plasticity and their spread to colder areas and the development of blooms under the scenario of global warming is predicted. Thus, the prevention and management of *Aphanizomenon* blooms will be a major challenge to scientists and water managers in future [11].

The genus *Aphanizomenon*, a filamentous cyanobacterium that belongs to the order Nostocales, has been known as the causative algae for cyanoHAB and most types of cyanotoxins including paralytic shellfish poisoning (PSP) toxins such as saxitoxins (SXTs), microcystins (MCs), cylindrospermopsins (CYNs) and anatoxins since the late 19th century and its occurrence rate has increased in recent decades [11,12,13]. It has also been reported that these species produce various secondary metabolites including off-flavor metabolites such as geosmin [14]. The type species of *Aphanizomenon* is *Aphanizomenon flos-aquae* (*A. flos-aquae*) Ralfs ex Bornet et Flahault 1888. In the past, most species were identified as *A. flos-aquae* and determined to be the causative species for the production of cyanotoxins [15,16,17]. However, detailed morphological classification and phylogenetic studies have reclassified *Aphanizomenon* spp. into various morphologically similar species such as *A. gracile* and *A. issatschenkoi,* rather than *A. flos-aquae* later [18,19,20]. Furthermore, recent report on potentially harmful *A. flos-aquae* in Korean river [21] indicates the geographic variability of intra-specific genotype. In addition, laboratory culturing may also change the original cyanobacterial morphological features making a correct identification even harder. Thus, it is necessary to use the polyphasic approaches by combining morphological and molecular methods based on the DNA sequence information of the genes to identify the causative cyanobacteria in blooms and their production potential for harmful secondary metabolites. 

The occurrence of the *Aphanizomenon* spp. cyanoHAB, which has the potential to produce harmful substances such as cyanobacterial toxins or off-flavor metabolites, in drinking water sources such as the Nakdong River [8,9,10], does not only call for an urgent response but also call for the critical verification of the harmful substances-producing potential of cyanoHAB-causing cyanobacteria. However, the identity of toxin producers can only be confirmed by isolating and identifying strains and by analytically confirming the presence of both the harmful metabolites and the genetic identity of the monoculture [11]. 

The present study aimed to precisely identify the causative cyanobacteria for cyanoHAB in the Nakdong River and evaluate their potential to produce harmful secondary metabolites to minimize the risk to both water quality and public health using molecular biology methods. Furthermore, it helps to select specific primer sets and PCR methods that can be used as monitoring tools for early detection of *A. flos-aquae* and cyanobacterial secondary metabolites.

## 2. Materials and Methods 

### 2.1. Sampling and Phytoplankton Analysis

**Figure 1:**
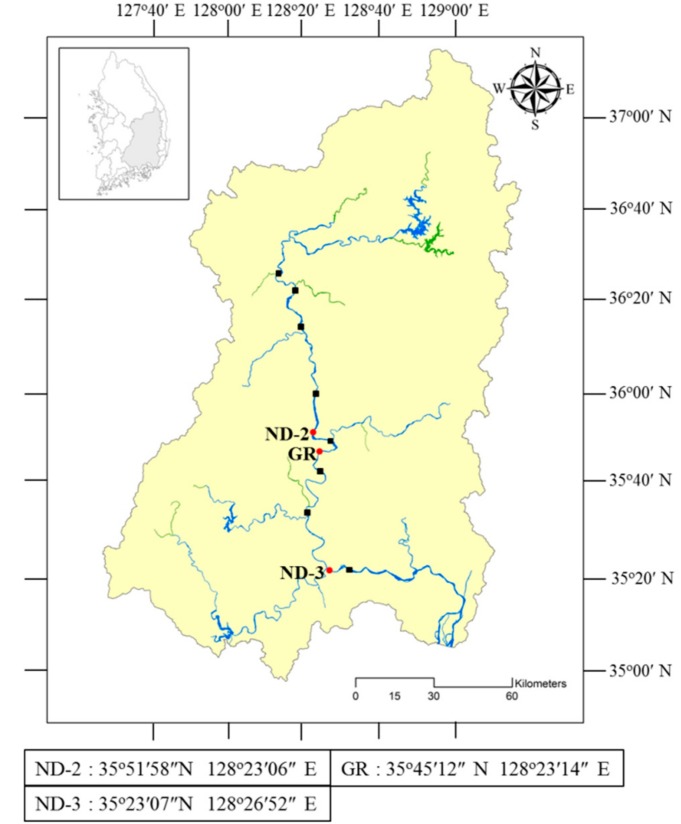
Map of the Nakdong River basin area. Red circles indicate the sampling stations and black squares indicate the weirs in the Nakdong River.

For molecular verification of cyanobacterial secondary metabolites production potential, surface water samples were collected monthly from two stations (ND-2, ND-3) in the Nakdong River, where the Harmful Cyanobacterial Bloom Alert System of the Nakdong River has been operating [22] from April to December, 2016 (Figure 1). The water samples were filtered through a 0.45 μm MicronSep nitrocellulose membrane disk (GVS Life Sciences, Findlay, OH, USA) and then stored in a cryogenic freezer (−80 °C) until molecular analysis. The water samples for phytoplankton analysis were preserved by adding Lugol’s solution (final concentration 0.3%). Phytoplankton was classified at the genus or species level under a microscope [23,24], counted using a Sedgwick-Rafter counting chamber and expressed as phytoplankton cell density as cells per mL of water.

### 2.2. Isolation and Culture of Aphanizomenon Strains

To isolate *Aphanizomenon* strains, cyanoHAB samples were collected at ND-2 and ND-3 on November 26 and 28, 2016 and at GR station on June 8, 2017 (Figure 1). The collected samples were examined under an optical microscope (Zeiss Axio Imager M1, Göttingen, Germany) using a cavity microscopic slide glass on which a droplet of CB liquid medium [25] for cyanobacteria was placed, and a colony of *Aphanizomenon* spp. was identified. The identified colonies were diluted with CB liquid medium until a single trichome was separated. The single trichome identified by the microscope was transferred to a culture bottle containing a CB liquid medium and cultured by shaking in an incubator at 20 °C with 40 μEm^−2^s^−1^ and L/D =14/10 h conditions. For each isolated strain, images were taken under the microscope, and cell size was measured, followed by subculture in a CB liquid medium every 4 weeks. To isolate genomic DNA from the *Aphanizomenon* isolates, the strains cultured in the CB liquid medium were filtered through a 0.45-μm MicronSep nitrocellulose membrane disk (GVS Life Sciences, Findley, OH, USA) and then stored in a cryogenic freezer (−80 °C) until use. 

### 2.3. Isolation of Genomic DNA 

Genomic DNA was isolated from cyanobacterial cells using the QIAGEN DNeasy Plant Mini Kit (QIAGEN, Hilden, Germany). A lysis buffer was added to filtered samples stored in the cryogenic freezer (−80 °C), followed by disruption of cells with a tissue grinder, and genomic DNA was extracted by centrifugation of the disrupted cells within the mini spin column. The concentration and purity of the DNA were measured using an Infinite M200 PRO Microplate Reader (Tecan Austria GmbH, Grödig, Austria). The isolated genomic DNA was stored at −20 °C.

### 2.4. Phylogenetic Analysis

For phylogenetic analysis of *Aphanizomenon* isolates, the nucleotide sequences of 16S rRNA and internal transcribed spacer region (ITS) between 16S rRNA and 23S rRNA were used. For sequence analyses of 16S rRNA and ITS region, CY16S-F1/CY16S-R2 and CY16S-F3/CY16S-R1 primers and APHITS-F/ATPITS-R primers were designed based on the sequence information of 16S rRNA and ITS in *A. flos-aquae* obtained from the GenBank database registered in the US NCBI (National Center for Biotechnology Information, NIH, Bethesda, MD, USA) (Table 1).

For amplification of the 16S rRNA and ITS region, PCR was performed at 95°C for 3 min, followed by 30 cycles of 95 °C for 30 s, 55 °C for 50 s and 72 °C for 1 min, and then treatment at 72 °C for 10 min, thus producing 840 and 763 bp PCR products for 16S rRNA and 312 and 541 bp PCR products for ITS. For nucleotide sequence analysis, the amplified PCR products were subjected to TA cloning using M13F and M13R primers, and then sent to Macrogen (Seoul, Korea) for sequencing. Multiple alignment of sequencing results with ClustalW in the BioEdit program resulted in a 1463 bp sequence for 16S rRNA and 266 and 495 bp sequences for ITS.

For phylogenetic analysis of 16S rRNA and 16S-23S rRNA ITS sequences, the sequence information of the 16S rRNA for *Aphanizomenon* spp., *Dolichospermum* spp., and *Cylindrospermopsis raciborskii* (*C. raciborskii*) and the ITS information of *Aphanizomenon* spp. were downloaded from the GenBank database of NCBI. Nucleotide sequences with a length of at least 1400 bp and 260 bp were selected for 16S rRNA and ITS, respectively, followed by ClustalW multiple alignment in the BioEdit program [32], which was used to construct a phylogenetic tree by the Neighbor-joining (NJ), Maximum likelihood (ML), and Maximum parsimony (MP) analyses using MEGA6 [33], with 1000 repetitions of bootstrap. For NJ, Kimura 2-parameter method [34] was used as a nucleotide substitution model. For ML, Kimura 2-parameter method with nearest-neighbor-interchange (NNI) heuristic search method was used. *Microcystis novacekii* (Accession No. AB035551) and *Microcystis aeruginosa* (Accession No. AB638217) was used as an outgroup to build 16S rRNA and 16S-23S rRNA ITS phylogenetic tree, respectively. 

### 2.5. Molecular Assays for Off-Flavor and Toxin Production

To evaluate the off-flavor-producing potential of field samples and *Aphanizomenon* isolates from the Nakdong River, the biosynthetic gene of geosmin (*geoA*) was analyzed. Some of the biosynthetic genes of MCs (*mcyA-Cd*, *mcyB*), SXTs (*sxtA*, *sxtI*) and CYNs (*cyrA*, *cyrJ*), were also analyzed. PCR was performed on the DNA extracts using the primer pairs presented in Table 1. The PCR reaction mixture was composed of 1.0 μL of DNA template, 12.5 μL of EmeraldAmp PCR Master Mix (Takara, Kusatsu, Japan), and 1 μL of primers (10 pmol) in a final volume of 25 μL. The PCR conditions for gene amplification were according to the protocols described by the authors (Table 1). 

The positive control used for verification of *geoA* gene was from *Dolichospermum planctonicum* (*D. planctonicum*, basionym *Anabaena planctonica*) NRERC-101 (Nakdong River Environment Research Center, NRERC, Goryeong, Korea), isolated from the Nakdong River, which had been confirmed to produce geosmin by instrumental analysis (gas chromatography/mass spectrometry, GC/MS) [35]. The positive control for *mcyA-Cd* and *mcyB* in the MC synthetase gene cluster was *M. flos-aquae* NRERC-205 (NRERC), which was isolated from the Nakdong River and had been confirmed to produce MCs through instrumental analysis (liquid chromatography/mass spectrophotometry, LC/MS). The genomic DNA of SXT-producing *A. gracile* NH-5 [18] was used as a positive control for *sxtA* and *sxtI* in the SXT gene cluster. The genomic DNA of CYN-producing *C. raciborskii* (CS-1101, Australian National Algae Culture Collection, ANACC, CSIRO, Clayton South, Australia) was used as a positive control for *cyrA* and *cyrJ* among the CYN gene cluster. The genomic DNA of *Escherichia coli* 5299 (Korean Collection for Type Culture, KCTC, Daejeon, Korea) and deionized water (DW) were used as negative controls and subjected to PCR amplification under the same condition used for amplification of each target gene. The size of the PCR products was checked by gel electrophoresis using 3% and 1.5% agarose gels and SiZer-50 (iNtRON, 24072) and SiZer-100 (iNtRON, 24073) DNA markers, respectively.

## 3. Results

### 3.1. Identification of Aphanizomenon Isolates by Morphological Characteristics

**Figure 2:**
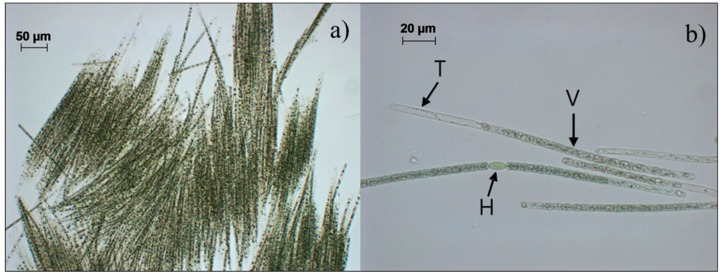
Microscope images of *Aphanizomenon flos-aquae* NRERC-006: (**a**) band-like fascicles, (**b**) single trichomes with vegetative cells (V), heterocytes (H), and typical apical cells (T).

Ten strains were isolated from CyanoHAB samples from the Nakdong River. Based on their morphological characteristics such as band-like fascicles with multiple trichomes, and almost hyaline apical cells, usually with characteristic remnants of cytoplasm in the form of an irregular lengthwise string, heterocytes solitary in a trichome, cylindrical, and akinetes, long cylindrical, distant from heterocytes [20], all strains were identified as *A. flos-aquae* Ralfs ex Bornet et Flahault 1888 (Figure 2, Table 2). All isolates were designated as NRERC-006, -007, -008, -009, -010, -011, -012, -013, -016 and -018 by the NRERC culture collection (NRERC, Goryeong, Korea). The size of vegetative cells of *A. flos-aquae* isolates in the present study was 4–10.6 × 3.5–5.8 μm.

### 3.2. Phylogenetic Analyses of 16S rRNA and ITS Sequences

For phylogenetic analysis using 16S rRNA sequences from the ten strains of *A. flos-aquae*, 1463 bp of the 16S rRNA sequences were obtained using the CY16S-F1/CY16S-R2 and CY16S-F3/CY16S-R1 primers designed in the present study, and the results were registered in the GenBank database of NCBI (accession numbers are listed in Table 2). 

BLAST analysis was performed with nucleotide sequences, including over 1400 bp from *Aphanizomenon* spp. and from similar species registered in the GenBank database of NCBI as well as the nucleotide sequences of the ten *A. flos-aquae* strains isolated in the present study. In the analysis, the ten isolates were classified as *A. flos-aquae*. They also showed over 99% sequence homology with 16S rRNA sequences from other *A. flos-aquae* strains (NCBI GenBank Accession No. KY327795, KY327796) isolated from the Nakdong River. Among the *A. flos-aquae* strains isolated in other countries, the 16S rRNA sequences of the present study showed 99% sequence homology with strains isolated from France (NCBI GenBank Accession No. AJ293123), Finland (AJ630441), and the UK (HE975013), and 98% sequence homology with those from Germany (KM019949), Lithuania (KM363406), and Thailand (AB862162). 

Overall branching pattern was similar in all the phylogenetic analyses (NJ, ML, MP). The strains isolated from the Nakdong River, including the *A. flos-aquae* isolates in the present study were supported by bootstrap values up to 80% and formed a single cluster. This was different from the clusters of *A. flos-aquae* or *A. gracile* isolated in other countries with 98~100% bootstrap support. In addition, *Dolichospermum* strains, *Anabaena* strains and *Sphaerospermopsis aphanizomenoides* (synonym, *Aphanizomenon aphanizomenoides*) were classified into a different group with >99% bootstrap support (Supplementary Material Figure S1).

**Figure 3:**
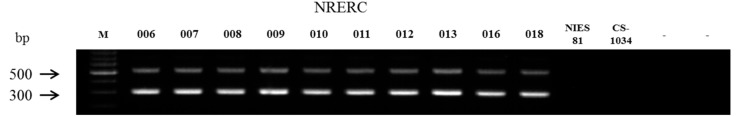
Gel electrophoresis image of ITS PCR products from *Aphanizomenon flos-aquae* NRERC–006 to NRERC–018, *Aphanizomenon gracile* strain NIES81, and *Chrysosporum ovalisporum* strain CS-1034. Upper bands show 541 bp ITS (type-2) PCR products and lower bands show 312 bp ITS (type-1) PCR products. M indicates SiZer-100 DNA marker.

In the present study, a primer set (APHITS-F/ATPITS-R primers) was designed to amplify the 16S-23S rRNA ITS region in the *A. flos-aquae* strains and to analyze its nucleotide sequence, which identified two characteristic types of bands with a size of 266 bp (type-1) and 495 bp (type-2) (Figure 3, Supplementary Material Table S1). The results were deposited in the GenBank database of NCBI (Table 2). It was previously reported that *A. flos-aquae* isolated in the Baltic Sea also had two ITS types of different sizes [36]. The ITS nucleotide sequences of the ten *A. flos-aquae* isolates in the present study showed 100% homology regardless of the collection site and date. However, when *A. gracile* (formerly *A. flos-aquae* f. *gracile*) NIES81 and *Chrysosporum ovalisporum* (*Ch. ovalisporum*, synonym *Aphanizomenon ovalisporum*) CS-1034 were subjected to PCR amplification under the same conditions, no PCR product was generated (Figure 3). 

**Figure 4:**
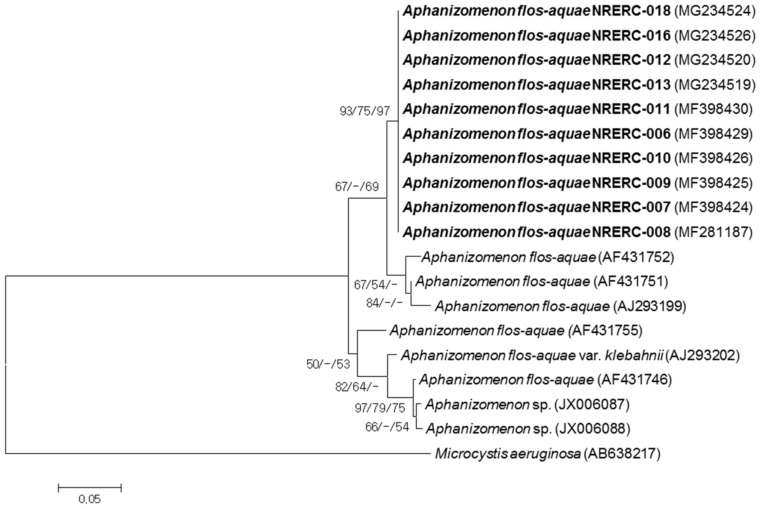
Neighbor-joining (NJ) tree of 16S-23S rRNA ITS sequences from *Aphanizomenon flos-aquae* strains. *Microcystis aeruginosa* (AB638217) was used as an outgroup. The numbers at the nodes indicate levels of bootstrap support (%) based on Neighbor-joining (NJ), Maximum likelihood (ML), and Maximum parsimony (MP) analysis of 1,000 resampled datasets. Only bootstrap values (NJ/ML/MP) above 50% are shown at the nodes. Strains of the present study are marked in bold and the GenBank accession numbers for ITS sequences are shown in parentheses. The scale bar indicates substitutions per site.

Of the two 16S-23S rRNA ITS types, the nucleotide sequence of ITS type-2, the longer ITS, was registered in the GenBank database of NCBI for the first time. Because the sequences of ITS type-2 of other *A. flos-aquae* isolates were not available in the database, we attempted to search for the ITS nucleotide sequence of *A. flos-aquae* in the GenBank database of NCBI, using the 266-bp DNA sequence of ITS type-1 as a query. To date, no ITS DNA sequence of *A. flos-aquae* isolated in Korean water systems is present in the GenBank database of NCBI except for that of the ten isolates in the present study. Hence, the ITS nucleotide sequences of *A. flos-aquae* strains isolated in Europe and the US were used to construct a phylogenetic tree using the NJ, ML and MP analyses. As a result, the *A. flos-aquae* strains isolated in the present study formed a cluster, and some strains isolated in Finland and France were separated as several clusters with >50% support (Figure 4). BLAST analysis found 89, 97, 94 and 93% sequence homology with ITS regions from *A. flos-aquae* strains isolated in Finland (AF431746, AF431751, AF431752, and AF431755, respectively), whereas there was 96% and 89% sequence homology with strains isolated in France (AJ293199 and AJ293202, respectively). In addition, there was 89% sequence homology with the strains from the US (JX006087 and JX006088).

### 3.3. Evaluation of Secondary Metabolite Production Potential 

**Figure 5:**
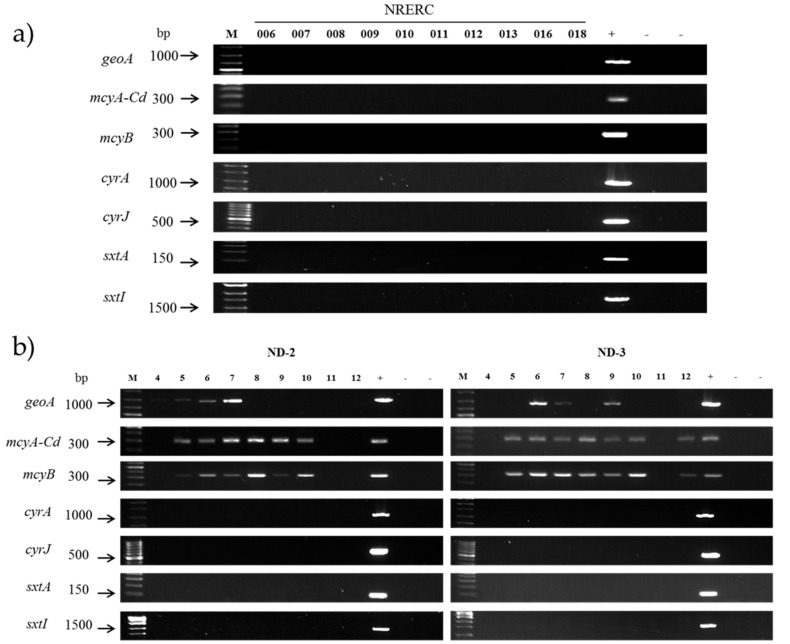
Gel electrophoresis image of the PCR products of the geosmin (*geoA*), microcystin (*mcyA-Cd*, *mcyB*), saxitoxin (*sxtA*, *sxtI*), and cylindrospermopsin (*cyrA*, *cyrJ*) biosynthesis genes from (**a**) *Aphanizomenon flos-aquae* isolates (NRERC–006 to NRERC–018) and (**b**) water samples. Sample number of ND-2 and ND-3 indicates the month of sampling date (Table 3). Positive (+) and negative (−) controls for each gene are described in the Materials and Methods section. M indicates SiZer-100 DNA marker.

To examine the potential of the *A. flos-aquae* strains to produce geosmin, PCR amplification was conducted with geo78F/geo982R primers for the *geoA* gene, but the 905 bp band was not found during electrophoresis. In contrast, PCR amplification using genomic DNA of *D. planctonicum* NRERC-101 resulted in the 905 bp band (Figure 5a). When the DNA sequences of the PCR product were BLAST searched in GenBank, they showed 99% homology with that of *Anabaena ucrainica* CHAB2155 (Accession No. HQ404997), confirming the presence of the geosmin synthase gene [35]. As a negative control, the genomic DNA of *E. coli* yielded no band in the corresponding size on electrophoresis. Therefore, it was confirmed that the ten *A. flos-aquae* strains isolated in the present study had no geosmin synthase gene.

The genomic DNA of the *A. flos-aquae* isolates was subjected to PCR using the *mcyA-Cd* and *mcyB* primer sets, which failed to amplify the 303 and 320 bp expected band. In contrast, PCR with positive control (*M. flos-aquae* NRERC-205) showed 303 and 320 bp PCR products on electrophoresis (Figure 5a). The PCR products of *mcyB* gene from *M. flos-aquae* NRERC-205 were sequenced and then searched against GenBank database using BLAST, which found 98% homology with the *mcyB* of *M. aeruginosa* UV027 (Accession No. AY034602) and *M. aeruginosa* PCC 7813 (Accession No. AY034601), indicating that the PCR product could be *mcyB*. Negative controls using *E. coli* and distilled water yielded no band on electrophoresis as in the *A. flos-aquae* isolates. From these results, it was confirmed that the ten *A. flos-aquae* isolates had no *mcyA-Cd* and *mcyB*. 

The genomic DNA of the *A. flos-aquae* isolates was subjected to PCR using the *sxtA* and *sxtI* primer set, which failed to amplify the 125- and 1669 bp expected bands as judged based on electrophoresis. In contrast, PCR amplification using genomic DNA of STX producing *A. gracile* NH-5 [17] under the same conditions produced the 125- and 1669 bp bands (Figure 5a), indicating that the ten *A. flos-aquae* isolates had no *sxtA and sxtI*.

To examine the presence of the CYN biosynthesis gene in *A. flos-aquae*, *cyrA* and *cyrJ* primer sets were used for PCR. The *A. flos-aquae* isolates failed to yield 1105–1179 bp and 578 bp bands that corresponded to the expected sizes of the PCR products. However, CYN-producing *C. raciborskii* CS-1101 produced 1105–1179 bp and 578 bp PCR products. The PCR products from *C. raciborskii* CS-1101 were sequenced and searched in GenBank database using BLAST. *CyrA* showed 99% homology with *cyrA* of *C. raciborskii* cyDB-1 (Accession No. KJ139707) and 96% homology with *aoa* gene cluster of *A. ovalisporum* (Accession No. AF395828). *CyrJ* showed 99% homology with *cyrJ* of *C. raciborskii* cyDB-1 (Accession No. KJ139693) and 94% homology with *cyrJ* of *A. ovalisporum* ILC-164 (Accession No. KJ139694). Therefore, it was confirmed that the ten *A. flos-aquae* isolates had no *cyrA* and *cyrJ*.

Monthly water samples from April to December in 2016 in the Nakdong River showed higher cyanobacterial cell density in the ND-3 station on the downstream than ND-2 station on the upstream (Table 3). *Aphanizomenon* spp. was present in all months in the range of 43–15,080 cells mL^−1^ in the ND-3 station. The cell density of *Microcystis* spp. was 18–71,625 cells mL^−1^ in the ND-2 and ND-3 stations but was not detected in April and November in both stations. *Dolichospermum* spp. showed the lowest cell density among the three genera (23–602 cells mL^−1^). PCR amplification for the geosmin synthase gene using genomic DNA of water samples yielded a 905 bp band in June and July in the ND-2 and ND-3 stations and September in the ND-3 station, corresponding to the months in which *Dolichospermum* spp. occurred in the river (Figure 5b). The PCR products were sequenced, searched in GenBank database using BLAST and identified as the geosmin synthase gene. All samples, positive for geosmin synthase gene, contained *Dolichospermum* spp. and *Microcystis* spp., but *Aphanizomenon* spp. presence did not correspond to the geosmin synthase gene. All samples, that did not yield PCR product did not contain *Dolichospermum* spp. regardless of *Microcystis* spp. and *Aphanizomenon* spp. cell density. Thus, the geosmin synthase gene of the samples might originate from co-occurring *Dolichospermum* spp. and not from *Aphanizomenon* spp.

PCR amplification with primer sets targeting partial regions of the MC synthetase gene cluster, the SXT gene cluster, and the CYN gene cluster using genomic DNA from water samples showed different results according to the target gene (Figure 5b). Samples collected from May to October showed the 303 and 320 bp band of the *mcyA-Cd* and *mcyB* PCR product in the ND-2 and ND-3 stations similar to the sample collected in December in the ND-3 station. All samples showing the *mcyA-Cd* and *mcyB* PCR product contained *Microcystis* spp., whereas samples without *mcyA-Cd* and *mcyB* PCR product did not contain *Microcystis* spp. The samples collected in ND-2 in May, July, August and October did not contain *Aphanizomenon* spp., whereas the samples collected in April in both stations and in November in the ND-3 station did not yield the *mcyA-Cd* and *mcyB* PCR product. Combined with the results of *Aphanizomenon* and *Microcystis* isolates, the *mcyA-Cd* and *mcyB* gene of the field samples might originate from co-occurring *Microcystis* spp. and not from *Aphanizomenon* spp. None of the water samples showed the expected band of PCR products of the *sxtA*, *sxtI*, *cyrA* and *cyrJ* primer sets on electrophoresis (Figure 5b) regardless of the cell density of major cyanobacterial genera. 

## 4. Discussion

*A. flos-aquae* is known to be the *Aphanizomenon* species blooming in the mainstream of the Nakdong River during spring and autumn [8,9]. Thus, the ten *Aphanizomenon* isolates, which were identified as *A. flos-aquae* by morphological and phylogenetic analysis, were confirmed as the *Aphanizomenon* species causing cyanoHAB in the Nakdong River. However, there were only 1–3 bp differences in the 16S rRNA nucleotide sequence and 100% sequence homology in the ITS among the ten strains isolated from three separate sites in spring and winter blooms, indicating a highly similar genotype of *A. flos-aquae* regardless of location and season of blooming in the Nakdong River.

The 16S rRNA gene is considered a molecular clock in the bacterial evolutionary process and is the most widely used genetic marker [11]. However BLAST analysis showed little variation in the 16S rRNA sequences of *A. flos-aquae*, regardless of geographical location. Increasing attention has been paid to the ITS region between 16S and 23S rRNA regions because it has a higher discerning power for the classification of allied species of cyanobacteria due to its high sequence variation [37]. In particular, the nucleotide sequence of rRNA ITS in cyanobacteria is an appropriate region to investigate intra-species variations [38]. The 89–97% sequence homology among the *A. flos-aquae* strains from the Nakdong River, Europe, and the US indicates higher intra-species variation of the nucleotide sequence in ITS regions than in 16S rRNA which showed 98–99% sequence homology among the *A. flos-aquae* strains. Thus, it is expected that the ITS region can be used to trace the geographical variations of *A. flos-aquae* nucleotide sequences. Additionally, the ITS primer set (APHITS-F/ATPITS-R) designed in the present study amplified the ITS nucleotide sequence in *A. flos-aquae*, but not those in *A. gracile* NIES81 and *Ch. ovalisporum* CS-1034 indicating this ITS primer set has potential to use molecular marker for *A. flos-aquae* molecular identification, but needs confirmation through the amplification with various *Aphanizomenon* species and other Nostocalean species.

The number of earthy/musty odor and taste episodes in treated water and customer complaints have increased worldwide, including Korea, during the last two decades [39,40]. Geosmin has been designated as a water quality surveillance indicator for drinking water in Korea. The occurrence of geosmin in the mainstream section of the Nakdong River, which serves as a drinking water source, is a significant issue, and determining the causative cyanobacteria can be the first step to control off-flavor substances. The level of geosmin in the Nakdong River is particularly high in spring [22]. During this season, the dominant cyanobacteria are mostly *Aphanizomenon* spp. and *Dolichospermum* spp., of which *Aphanizomenon* spp. has a higher dominance and has recently began blooming from autumn to early winter, in addition to spring [9,10]. Hence, if *Aphanizomenon* spp. possess the geosmin synthase gene, it will likely increase the level of off-flavor in the water. However, between *Aphanizomenon* spp. and *Dolichospermum* spp. isolated from the Nakdong River, the present study found the geosmin synthase gene only in *Dolichospermum* spp. and not in *Aphanizomenon* spp. Moreover, the geosmin synthase gene was amplified in the field samples with *Dolichospermum* spp. and not in the field samples with only *Aphanizomenon* spp. (April in the ND-2 and ND-3, November in the ND-3) Therefore, it appears that *Dolichospermum* spp. cause geosmin contamination in the Nakdong River and that there should be a low potential for off-flavor during the blooms of *A. flos-aquae*.

The genus *Aphanizomenon* comprises a number of cyanoHAB, producing cyanotoxins in water bodies worldwide [11]. At present, MCs are the most representative cyanobacterial toxins that have been listed in the drinking water guidelines published by the WHO, and MC-LR has been designated as a water quality surveillance indicator for drinking water in Korea. It has been identified worldwide that MCs are produced by cyanobacteria, including *A. flos-aquae*, that cause harmful cyanobacterial blooms [1]. The production of microcystins and the presence of *mcyA* gene in the *A. flos-aquae* in Korea has also been reported [21]. However, considering the results of the present study, it seems that *A. flos-aquae*, which is dominant and blooms in the Nakdong River during spring, autumn and early winter, has low potential to produce MCs.

*SxtA* encodes polyketide synthase which initiates STX biosynthesis, and it has been detected not only in STX producing strains, but also in STX non-producing strains similar to *sxtG*, *sxtH* and *sxtX* [41]. In contrast, *sxtI* has been detected only in STX-producing strains to date in genus *Aphanizomenon* with exception in genus *Cylindrospermopsis* and *Lyngbya* [42,43]; therefore, it was suggested as a marker for verification of STX-producing *Aphanizomenon* strains [11]. To date, various filamentous cyanobacterial species including *A. gracile*, *Cuspidothrix issatschenkoi*, *C. raciborskii*, *D. circinale*, and *Geitlerinema* spp. have been identified to produce PSP toxins including STX, whereas toxin production by *A. flos-aquae* is controversial [11]. Since the late 19th century, *A. flos-aquae* has been reported as a potential producer of cyanobacterial toxins, and it was reported in 1967 for the first time that *A. flos-aquae* produces a toxin with a structure similar to that of STX produced by marine dinoflagellates [44]. Although *A. flos-aquae* was identified as a producer of STX in the US in 1980 [16], morphological and genetic re-evaluation subsequently revealed that it was not *A. flos-aquae*, but *A. gracile* [18]. Thus, it remains unclear whether *A. flos-aquae* produces PSP toxins. Nevertheless, given that neither *sxtA* nor *sxtI* of SXT biosynthesis gene cluster was confirmed in the *A. flos-aquae* strains isolated in the present study or in the field samples from ND-2 and ND-3 stations containing *Aphanizomenon* spp., there likely is a low potential of STX occurrence in *A. flos-aquae* blooms in the surveyed stations of the Nakdong River during spring, autumn, and winter.

Cylindrospermopsin (CYN) is a cytotoxin reported to occur mostly at high water temperature and was first identified to be produced by *C. raciborskii*, which mostly blooms in tropical water bodies; however, it has also been reported in field samples or isolates from Oceania, Asia, America, and Europe [45,46]. The most representative CYN-producing cyanobacteria is *C. raciborskii*; however, CYN is also known to be produced by major species in the order Nostocales, including *Ch. ovalisporum*, *A. flos-aquae*, *Anabaena bergii* and *Raphidiopsis curvata* as well as *Umezakia natans* (order Stigonematales) and *Lyngbya wollei* (order Oscillatoriales) [43]. Thus, it is critical to investigate whether CYN is present in the Nakdong River, where *A. flos-aquae* is dominant. In the CYN gene cluster, sulfotransferase genes are present in only CYN-producing strains. Thus, *cyrJ* should be a useful marker for CYN toxicity, because it encodes adenylyl sulfate kinase, which is an enzyme catalyzing the biosynthesis of 3′-phosphoadenosine-5′-phosphosulfate (PAPS), the sulfate donor of sulfotransferase [47]. Therefore, given that the PCR product of *cyrJ* was not detected in the *A. flos-aquae* isolates in the present study or in field samples containing *Aphanizomenon* spp., the *A. flos-aquae* in the Nakdong River should have low potential to produce CYN.

## 5. Conclusions

In this study, ten *A. flos-aquae* strains were isolated from the cyanoHAB in the Nakdong River, which were identified by using morphological and phylogenetic analysis and show highly similar genotype regardless of location and season of blooming in the Nakdong River. 89–97% sequence homology among the *A. flos-aquae* strains from the Nakdong River and Europe and the US indicates high intra-species variation of the nucleotide sequence in ITS, which can be used to trace the geographical variations of *A. flos-aquae* nucleotide sequences.

We further verified the potential of cyanoHAB-causing *A. flos-aquae* strains and water samples containing cyanobacterial species in producing prominent harmful secondary metabolites using PCR methods with specific primer sets and all isolates and the water sample containing only *Aphanizomenon* spp. had a low potential to produce these metabolites. However, water samples containing other cyanobacterial species such as *Microcystis* and *Dolichospermum* spp. possessed *mcyA* and *mcyB* of the MC synthetase gene cluster and the geosmin synthase gene respectively. Therefore, bloom-forming cyanobacterial strains in addition to *Aphanizomenon* spp. isolated during other times and sites as well as field samples collected during the time when such cyanobacteria are dominant should be continuously monitored for the presence of the harmful secondary metabolites and their biosynthesis genes to minimize the risk to both water quality and public health. The PCR assay with the specific primer sets of this study is expected to provide a rapid and sensitive means for early detection of *A. flos-aquae* and potentially harmful populations of cyanobacteria, especially in the initial stage of cyanobacterial growth in water supplies.

## Figures and Tables

**Table 1:** List of primers used in this study.

Target Gene	Primer Set	Sequence (5′—3′)	Fragment Length (bp)	References
*16S rRNA*	CY16S-F1	CAGGATGAACGCTGGCGG	840	This study
	CY16S-R2	GAATGGGATTAGATACCCCA		
	CY16S-F3	AAAGGAGGTGATCCAGCCAC	763	This study
	CY16S-R1	TCCTTTGAGTTTCACAGTTG		
*ITS*	APHITS-F	ACAAGGTAGCCGTACCGGAA	312/541	This study
	APHITS-R	ACAATTTCTTTTGCTTCCAC		
*geoA*	geo78F	GCATTCCAAAGCCTGGGCTTA	905	[14]
	geo982R	ATCGCATGTGCCACTCGTGAC		
*mcyA-Cd*	mcyAF	AAAAGTGTTTTATTAGCGGCTCAT	303	[26]
	mcyAR	AAAATTAAAAGCCGTATCAAA		
*mcyB*	mcyB2959F	TGGGAAGATGTTCTTCAGGTATCCAA	320	[27]
	mcyB3278R	AGAGTGGAAACAATATGATAAGCT AC		
*cyrA*	CYLAT-F	ATTGTAAATAGCTGGAATGAGTGG	1105–1179	[28]
	CYLAT-R	TTAGGGAAGTAATCTTCACAG		
*cyrJ*	cynsulfF	ACTTCTCTCCTTTCCCTATC	578	[29]
	cylnamR	GAGTGAAAATGCGTAGAACTTG		
*sxtA*	sxtA-F	GATGACGGAGTATTTGAAGC	125	[30]
	sxtA-R	CTGCATCTTCTGGACGGTAA		
*sxtI*	sxtI-F	GCTTACTACCACGATAGTGCTGCCG	1669	[31]
	sxtI-R	GGTTCGCCGCGGACATTAAA		

**Table 2:** Information for *Aphanizomenon flos-aquae* strains isolated from the Nakdong River and NCBI GenBank Accession Numbers of the 16S rRNA and 16S-23S rRNA ITS sequences of each strain.

Strain No. (NRERC-)	Scientific Name	Collection Date	Isolation Source	NCBI GenBank Accession Number		
				16S rRNA	ITS Type-1	ITS Type-2
006	*Aphanizomenon flos-aquae*	26 November 2016	ND-2	MF362173	MF398429	MF398428
007	*Aphanizomenon flos-aquae*	28 November 2016	ND-3	MF362174	MF398424	MF398427
008	*Aphanizomenon flos-aquae*	26 November 2016	ND-2	KY952630	MF281187	MF322523
009	*Aphanizomenon flos-aquae*	28 November 2016	ND-3	MF362175	MF398425	MF398423
010	*Aphanizomenon flos-aquae*	28 November 2016	ND-3	MF362177	MF398426	MF398422
011	*Aphanizomenon flos-aquae*	26 November 2016	ND-2	MF362176	MF398430	MF422681
012	*Aphanizomenon flos-aquae*	28 November 2016	ND-3	MG234522	MG234520	MG234525
013	*Aphanizomenon flos-aquae*	28 November 2016	ND-3	MG234521	MG234519	MG234527
016	*Aphanizomenon flos-aquae*	8 June 2017	GR	MG234523	MG234526	MG234528
018	*Aphanizomenon flos-aquae*	8 June 2017	GR	MG234518	MG234524	MG234529

**Table 3:** Cell densities (cells mL^−1^) of three major cyanobacterial genera of monthly water samples from the Nakdong River.

Sampling Date	ND-2			ND-3		
	*Aphanizomenon* spp.	*Microcystis* spp.	*Dolichospermum* spp.	*Aphanizomenon* spp.	*Microcystis* spp.	*Dolichospermum* spp.
25 April 2016	19	ND	ND	43	ND	ND
30 May 2016	ND	1525	ND	700	17,280	ND
20 June 2016	600	1868	314	15,080	7143	602
25 July 2016	ND	12	23	365	3630	375
22 August 2016	ND	3270	ND	2184	71,625	ND
26 September 2016	35	83	ND	202	89	80
24 October 2016	ND	18	ND	64	51	ND
28 November 2016	ND	ND	ND	1532	ND	ND
26 December 2016	ND	ND	ND	227	50	ND

## Supplementary Materials

The following are available online at http://www.mdpi.com/1660-4601/15/8/1739/s1, Figure S1: Neighbor-Joining (NJ) tree of 16S rRNA sequences from *Aphanizomenon flos-aquae* and Nostocales cyanobacterial strains, Table S1: Nucleotide sequence of 16S-23S rRNA ITS of *Aphanizomenon flos-aquae* isolated from the Nakdong River.
