# Supplementary material for: Molecular Verification of Bloom-forming Aphanizomenon flos-aquae and Their Secondary Metabolites in the Nakdong River
Source: Int J Environ Res Public Health. 2018 Aug 13;15(8):1739. doi: 10.3390/ijerph15081739 (PMC6121560; doi:10.3390/ijerph15081739)
Supplement: Supplementary file 1 [file ijerph-15-01739-s001.pdf]

Supplementary Material

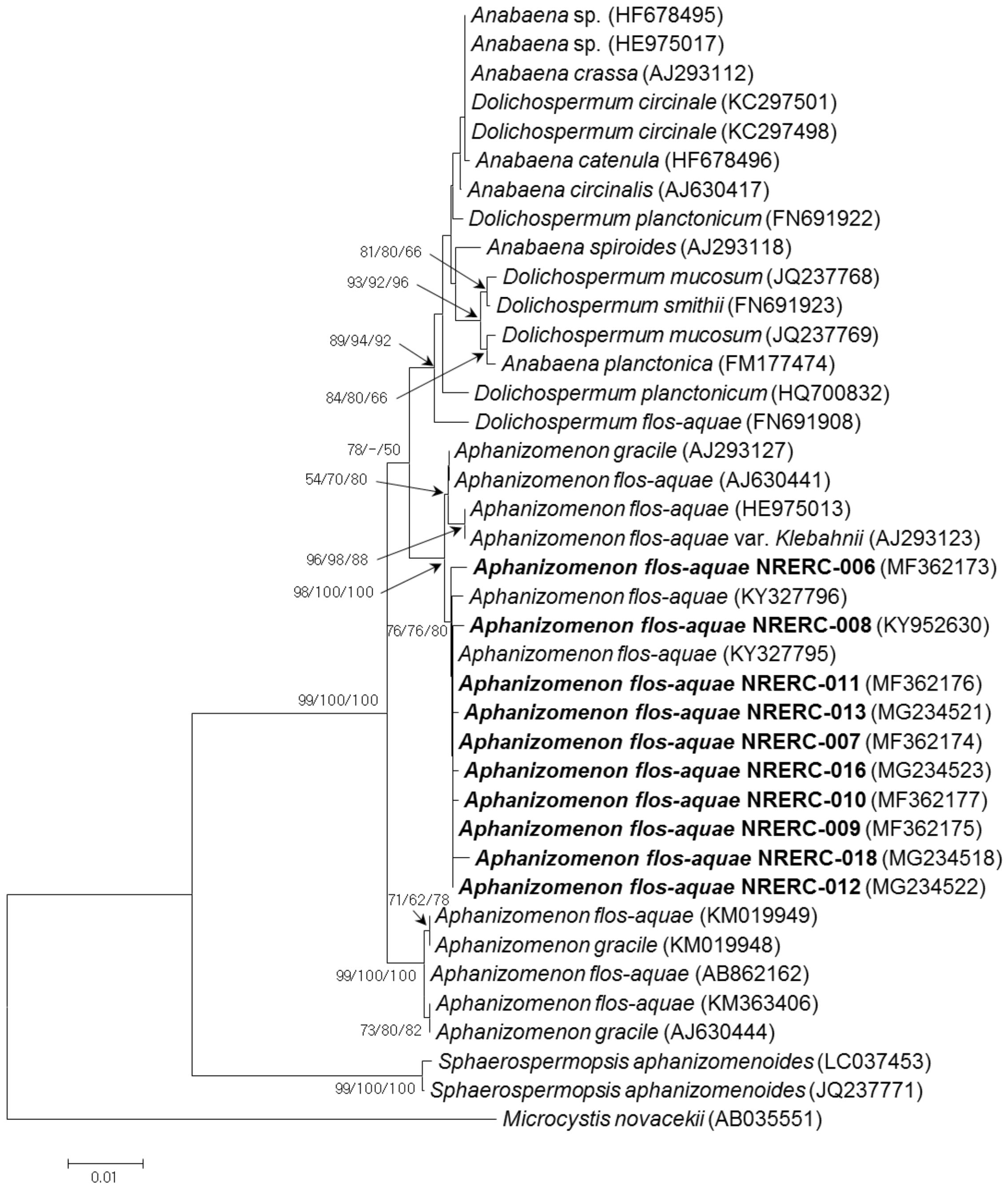

Figure S1. Neighbor-Joining (NJ) tree of 16S rRNA sequences from *Aphanizomenon flos-aquae* and Nostocales cyanobacterial strains. *Microcystis novacekii* (AB035551) was used as an outgroup. The numbers at the nodes indicated levels of bootstrap support (%) based on Neighbor-Joining (NJ), Maximum likelihood (ML), and Maximum parsimony (MP) analysis of 1,000 resampled datasets. Only bootstrap values (NJ/ML/MP) above 50% are shown at the nodes. Strains of the present study are marked in bold and the GenBank accession numbers for 16S rRNA gene sequences are shown in parentheses. The scale bar indicates substitutions per site.

Table S1. Nucleotide sequence of 16S-23S rRNA ITS of *Aphanizomenon flos-aquae* isolated from the Nakdong River. Sequence direction is 5′ to 3′.

Type-1		
1	TAGGGAGACCTAATCCATTTAGAAATCGAAGGTGATGAAAATTGCCAATA	50
51	GAAACTAAATTGGTCTAACCTAGGTCGGTCGAGATTGGGAAAAATCTTTC	100
101	AAAGTATTATTTCGGCTTAGTTAAATAGGAAAAAAGACAGCACCTAACTA	150
151	TAAAAGTTAGAATGCTGGATGAAATTCCAGTCAGAACCTTGAAAACTGCA	200
201	TAAGAGAAAGAGAAAAAGCACCGCAGACACAGACATTTAGATGGATGTGG	250
251	AAGCAAAAGAAATTGT	266
Type-2		
1	TAGGGAGACCTAATCCATTTAGAAATCGAGGGTGATGAAAATTGCTAATA	50
51	GAAACTAAATCGGTCTAACCTAGGTCGGTCGCAGACTTGAAGTAAAGCTT	100
101	TCAAACTATGATTTGGTTCGATAAGGGCTATTAGCTCAGGTGGTTAGAGC	150
151	GCACCCCTGATAAGGGTGAGGTCCCTGGTTCGAGTCCAGGATGGCCCACC	200
201	TGAAGGAAGTCAAAAATCAAAAGTCAAAAGTCAAAAGTAAATATTTTTGA	250
251	GCCGTGACTATAAATTTTGAGTTCTTTTTGGGGGTTTAGCTCAGTTGGTA	300
301	GAGCGCCTGCTTTGCAAGCAGGATGTCAGCGGTTCGAGTCCGCTAACCTC	350
351	CACATTGGAAAAAAGACAGCACCTAACTATAAAAGTTAGAATGCTGGATG	400
401	AAATTCCAGTCAGAACCTTGAAAACTGCATAAGAGAAAGAGAAAAAGCAC	450
451	CGCAGACACAGACATTTAGATGGATGTGGAAGCAAAAGAAATTGT	495
